# High spatial variation in population size and symbiotic performance of *Rhizobium leguminosarum* bv. *trifolii* with white clover in New Zealand pasture soils

**DOI:** 10.1371/journal.pone.0192607

**Published:** 2018-02-28

**Authors:** Steven Wakelin, Guyléne Tillard, Robert van Ham, Ross Ballard, Elizabeth Farquharson, Emily Gerard, Rene Geurts, Matthew Brown, Hayley Ridgway, Maureen O’Callaghan

**Affiliations:** 1 AgResearch Ltd, Lincoln Science Centre, Christchurch, New Zealand; 2 Institut National Superieur des Sciences Agronomiques, Dijon, France; 3 Laboratory of Molecular Biology, Wageningen University, Wageningen, The Netherlands; 4 South Australian Research and Development Institute, Urrbrae, South Australia, Australia; 5 Faculty of Agriculture and Life Sciences, Lincoln University, Christchurch, New Zealand; National Cheng Kung University, TAIWAN

## Abstract

Biological nitrogen fixation through the legume-rhizobia symbiosis is important for sustainable pastoral production. In New Zealand, the most widespread and valuable symbiosis occurs between white clover (*Trifolium repens* L.) and *Rhizobium leguminosarum* bv. *trifolii* (*Rlt*). As variation in the population size (determined by most probable number assays; MPN) and effectiveness of N-fixation (symbiotic potential; SP) of *Rlt* in soils may affect white clover performance, the extent in variation in these properties was examined at three different spatial scales: (1) From 26 sites across New Zealand, (2) at farm-wide scale, and (3) within single fields. Overall, *Rlt* populations ranged from 95 to >1 x 10^8^ per g soil, with variation similar at the three spatial scales assessed. For almost all samples, there was no relationship between rhizobia population size and ability of the population to fix N during legume symbiosis (SP). When compared with the commercial inoculant strain, the SP of soils ranged between 14 to 143% efficacy. The N-fixing ability of rhizobia populations varied more between samples collected from within a single hill country field (0.8 ha) than between 26 samples collected from diverse locations across New Zealand. Correlations between SP and calcium and aluminium content were found in all sites, except within a dairy farm field. Given the general lack of association between SP and MPN, and high spatial variability of SP at single field scale, provision of advice for treating legume seed with rhizobia based on field-average MPN counts needs to be carefully considered.

## Introduction

Legumes, in symbiosis with rhizobia bacteria, can fix atmospheric nitrogen into plant available forms [1]. Over time, biological nitrogen fixation (BNF) increases the fertility and productive capacity within natural and managed ecosystems [2]. In New Zealand, where pastoral agriculture is extensively used to support livestock production, BNF supplied from the legume-rhizobia symbiosis drives growth of non-leguminous forages species such as grasses and herbs [3]. Furthermore, BNF inputs of N into farming systems from can have a lower environmental cost compared with chemical N fertilisers [3]. Thus, effective legume-rhizobia symbiosis can support the productivity and profitably of pastoral farming, whilst reducing the environmental footprint [4].

Many factors can affect legume establishment and growth in agricultural soils. Constraints can include hostile soil conditions (e.g. pH, high Al^3+^ content, P-infertility), adverse climatic conditions (soil moisture deficit, extremes of temperature), through to pests, diseases, and weeds (see review by Tozer et al. 2013 [5] and references therein). In many cases, these can be addressed through careful selection of legume species that match the soil and environmental conditions, and effective management of the farming system such as timing of over-sowing and controlling of grazing pressure [5]. However, the success of legumes in agricultural systems is also affected by the availability of sufficient population sizes of nodule-forming (nod^+^) and nitrogen-fixing (fix^+^) rhizobia in soils [6,7]. These not only determine if legumes can enter nodule-forming symbioses, but also how effectively the symbiosis can fix nitrogen.

White clover (*Trifolium repens* L.) is the most widely grown pasture legume in New Zealand [4]. The species is phenotypically diverse and cultivars suited to many grazing and management systems are available [8]. An agronomic strength of white clover is its phenotypic plasticity, underpinned by its high genetic diversity arising from being an obligate out-crosser [9]. This gives rise to locally-selected populations that can be adapted to a range of different pastoral agroecosystems [10].

The rhizobia capable of forming root nodule symbiosis with white clover (*Rhizobium leguminosarum* bv. *trifolii*; *Rlt*) were likely introduced into New Zealand on soil and plant material during European settlement in the early 20^th^ century [11]. However, occurrences of white clover establishment failure occurred, particularly during large scale pasture development on land cleared of native forest/tussock grassland. This led to recommendations to inoculate white clover seed with *Rlt*. Since the 1960s., effective *Rlt* strains (mostly sourced from Australia) have been available to farmers [11].

As a result of both deliberate inoculation and accidental introduction, *Rlt* populations became widespread in New Zealand’s agricultural soils; populations of >100,000 clover rhizobia per gram of soil have been measured in many studies [11–14]. However, while rhizobia can be plentiful in some soils, early work also showed that these naturalised populations vary in symbiotic potential, i.e. their effectiveness at fixing N_2_ with a host legume relative to a selected control strain (generally a strain used commercially). For example, a survey of rhizobia naturalised in Otago soils found N_2_-fixation ability differed between 2 and 118% when compared with a commercial inoculant strain (CC275e/PDDCC 2163; [13,15]). Similar levels of variability in effectiveness were reported in multiple other studies [11]. However most of this work was conducted using sampling strategies that pool sub-samples of soil collected within a field. As such, the magnitude of variation in population size and symbiotic potential within a field, as compared to between fields, is unknown. A high field-average value may, therefore, lead farmers to conclude that soil rhizobia resources are present to support clover production. However this value masks the presence of localised areas with insufficient rhizobia numbers or populations of symbiotic performance, giving rise to sub-optimal production. To mitigate against this, there may be merit in treating all seed with rhizobia (insurance inoculation). This is a key question for countries such as New Zealand which rely on agricultural legume production.

This study examines variability in size and effectiveness of naturalised *Rlt* populations in pasture soils. The work was conducted over different spatial scales, with the aim of determining if factors associated with the size and effectiveness of rhizobia at broad geographic scale are similar to those within single fields. Finally, we aimed to determine the extent of association between rhizobia population size and nitrogen fixation ability of white clover in New Zealand pastures.

## Methods and materials

### Sites and sampling

Variation in the size and effectiveness of white clover nodulating (nod^+^) *Rlt* populations in New Zealand pasture soils was assessed at three spatial scales: (1) across New Zealand, (2) at large-farm scale, and (3) within-field variation for a hill country sheep and beef farm, and also a dairy farm with relatively flat topography. All samples were collected from privately owned land; consent was provided from farmers at the time of collection.

**Fig 1:**
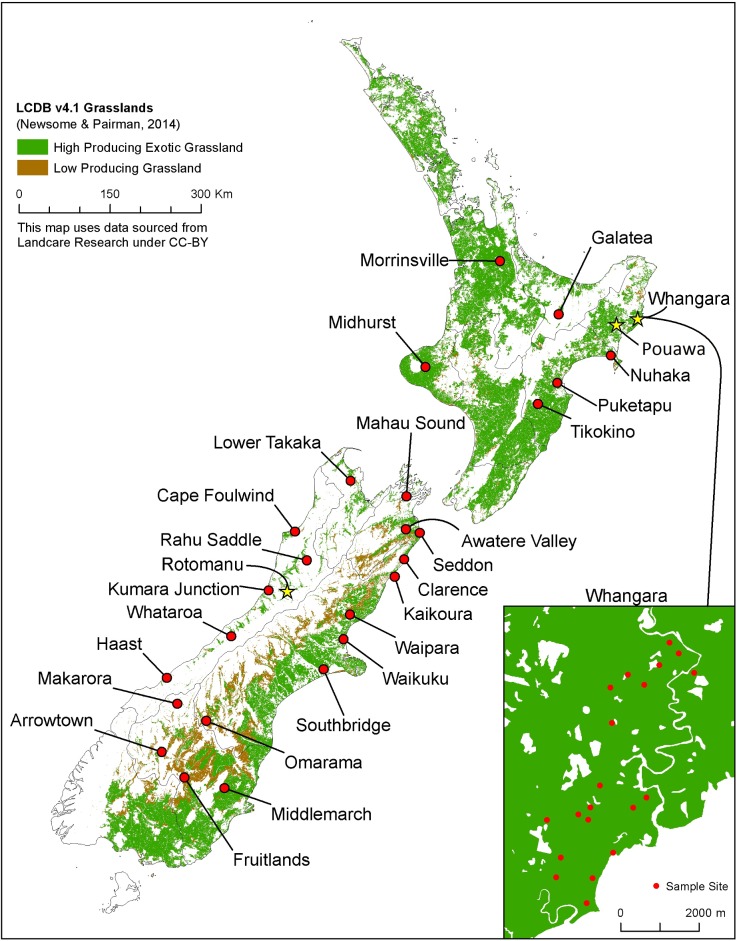
Sampling sites from across New Zealand and the Whangara farm (call out box). The Pouawa and Rotomanu sites, where spatially-dense sampling was conducted a single field, are labelled (star symbols). Location data (GPS) and soil type information can be found in the Supplementary Information, S1 Table.

The New Zealand wide sampling was undertaken on 26 properties; these are shown in Fig 1. Site descriptions and soil sampling techniques for these sites been reported previously [16]. Briefly, the farms ranged from low through to high intensity farming systems. However, all soil samples were collected from fields that been continuously engaged in livestock-grazed pastoral production for a decade or longer. From each site, >40 individual soil were taken to 10 cm depth using a soil corer (2.5 cm diameter). Samples were taken from one site on the farm and were pooled into a composite sample of approximately 3 kg. On return to the laboratory, the soil cores were thoroughly mixed and stored at 4°C for further analysis.

At farm scale, samples were collected from 20 fields at ‘Whangara’, an extensive 7,100 ha coastal hill country farm north of Gisborne (Fig 1; S2 Table). A sample was collected from a single location in each field, with approximately 3 kg of material collected to a depth of 10 cm. The distances between sampling points (fields) varied from 300 metres to 7 km, with an average distance of 2.5 km between sample collection points (Fig 1).

Within paddock variation was assessed at two sites selected to represent contrasting farming operations: ‘Pouawa’, a hill-country sheep and beef farm west of Gisborne, and ‘Rotomanu’, a dairy farm near Lake Brunner (Fig 1; S3 Table). From within a single field at each location, fifty samples were collected in a systematic sampling pattern. At Pouawa, this was a 0.8 ha, SW-facing hill face with a 15 m drop in elevation from the highest to lowest samples. At Rotomanu, 50 samples were collected from within a 1.8 ha field with flat topography using the same sampling approach (S4 Table). At each sampling point, approximately 3 kg of soil was collected to 10 cm depth.

### Soil physicochemical analysis

Physicochemical properties of the soils were determined by Hill Laboratories (Hamilton, NZ). Analyses included: pH, Olsen P, K, Ca, Mg, Na, available N, anaerobically mineralisable N (AMN), Al_ex_ (i.e. CaCl_2_ extractable), total C, total N, C:N, total P, ammonium N (${NH}_{4}^{+}$), nitrate N (${NO}_{3}^{-}$), total mineral N, cation exchange capacity (CEC), and total base saturation (TBS). Details of the soil processing and analytical methods are provided in full elsewhere [16,17], or can be found on the provider’s website [18]. The physicochemical data for all soil samples can be found in S1–S4 Tables.

### Meteorological data

For the samples collected from across New Zealand, the GPS location data was used to extract environmental data based on the virtual climate station network. This provides an interpolation-derived estimate calculated from empirical data observations from actual climate stations [19]. These were used to determine average daily maximum and minimum temperature, rainfall, and solar radiation (rad) [20], and the data was averaged for the 2004–2014 period. Meteorological data was not collected or used for the other study sites, as variation in these variables at farm or field scale would be minimal and variation in rhizobia population values would be essentially compared to single values.

### Soil rhizobia population size

Populations of *Rlt* in soils were measured using the host-baiting and most probable number (MPN) technique [21] as described previously [16]. Briefly, from the pooled soil sample from each site, a ten-fold dilution series (10^−1^ to 10^−6^) was prepared and aliquots (100 μl) from each dilution used to inoculate five replicate tubes containing a surface sterilised white clover seed (cv. Tribute) along with N-free growth medium (vermiculite and N-free McKnight’s solution). The tubes were covered and maintained under controlled environmental conditions for 6 weeks. Seedlings were then removed from tubes and examined for the presence of nodules. Numbers of *Rlt* were calculated using the MPN calculator [22,23]; all data are expressed as log_10_ of the number of *Rlt* per g (dry weight) of soil.

### Soil rhizobia population symbiotic potential

Nitrogen fixation capacity of the symbiosis (SP) was compared against white clover growth when inoculated with *Rlt* strain TA1. Strain TA1 was used as a reference point for ‘100% effective’ as it is the current commercially-available inoculant for white clover seed in New Zealand [11]. Symbiotic potential of the rhizobia from the soil samples was determined using growth of *T*. *repens* cv. Tribute under N-limited conditions. As such, plant growth is in direct relationship with N-fixation provided through the *Rlt* symbiosis. Clover seeds were surface sterilised by immersion in 70% ethanol for 60 s, 4% NaHClO_4_ (w/v) for 30 s, and then washed six times with sterile H_2_O. Sterilised seeds were individually placed within layers of sterile damp paper towels, incubated for 24 h at 4°C, and then overnight at 20°C to germinate. Germinated seeds were planted (one plant per pot) into vermiculite (Grade 2, Exfoliators (Aust) Pty Ltd) that had been firmly packed into 70 ml plastic containers. The containers were moistened with 40 ml of nutrient solution (McKnight’s solution; [24]) containing trace N (0.1 mM NH_4_NO_3_), and sterilised by autoclaving at 121°C for 10 min.

Plants were inoculated 3 days after sowing. Soil samples (10 g) were diluted in 90 ml of 0.85% NaCl solution, shaken for 15 min, and 1 ml of the soil suspension was applied to each plant. The reference strain TA1 was grown in yeast mannitol broth (mannitol 10 g, K_2_HPO_4_ 0.5 g, MgSO_4_·7H_2_O 0.2 g, NaCl 0.1 g, yeast extract 1.0 g, distilled water 1 L) for 48 h at 28°C. Cells were harvested by centrifugation, resuspended in 0.85% NaCl, diluted to 1.5 x 10^4^ cells per ml, and applied at 1 ml per plant. Uninoculated treatments received 1 ml sterile saline. Positive N controls received 1 ml of 10 mM NH_4_NO_3_ solution 7 days after inoculation, then on every seventh day until harvest. Each treatment had eight replicate pots. These were arranged in a randomised block design in a growth room (16 h light at 22°C; 8 h dark at ambient temperature) and watered with sterile water as required.

Plant shoots were harvested 42 days after inoculation, dried at 60°C for 48 hours, and shoot dry weights (SDW) recorded. The symbiotic potential of each soil rhizobial population was calculated as described in Drew et al. [25], using the equation below which accounts for differences in dry matter production associated with seed N and any nutrients added in soil suspensions:

$$Symbiotic\;potential=\frac{{(SDW}_{inoc}-{SDW}_{uninoc})}{{(SDW}_{TA1}-{SDW}_{uninoc})}\times 100$$

where subscripts “inoc” indicate where plants are inoculated with soil suspensions containing rhizobia populations, “uninoc” where plants remain uninoculated, and ‘TA1’ where plants are inoculated with a known concentration of the current commercial strain.

### Data analysis

For samples collected at each spatial scale (New Zealand wide, across farm, and within paddock), linear regression was used to test for association between rhizobia population size and effectiveness (SP). Differences in average MPN and SP among sample sets were assessed using Kruskal-Wallis one-way analysis of variance across the sample sets, with Bonferroni’s multiple comparisons test used to determine pair-wise effects. These analyses were conducted in GenStat 17 (VSN International Ltd).

Correlations (Pearson’s) between the soil physicochemical properties with MPN and SP were conducted to determine underlying relationships between rhizobia populations and site characteristics. For the New Zealand wide data, analysis included the environmental variables as described previously. These analyses were conducted in Prism 7.03 (GraphPad Software Ltd).

To determine if the variation in MPN and SP data was different when sampling was conducted at different spatial scales, an equality of variance test was conducted (Levene’s test) in the CAR package [26].

Variation at farm and field scales were mapped in ArcGIS Desktop (Environmental Systems Research Institute, Inc.) and a number of interpolation techniques were evaluated using the Geostatistical Analyst extension. The thin-plate spline radial basis function was determined to be more suitable than Kriging, especially for the Pouawa and Rotomanu sites due to the ability to create a smoothed surface passing through each measured sample. The distribution of the sample points and the complexity of the Whangara site made the assessment of the prediction surfaces less straightforward so the thin-plate spline technique was used for consistency across all sample sets.

## Results

### Numbers of *R*. *leguminosarum* bv. *trifolii* in pasture soils

**Fig 2:**
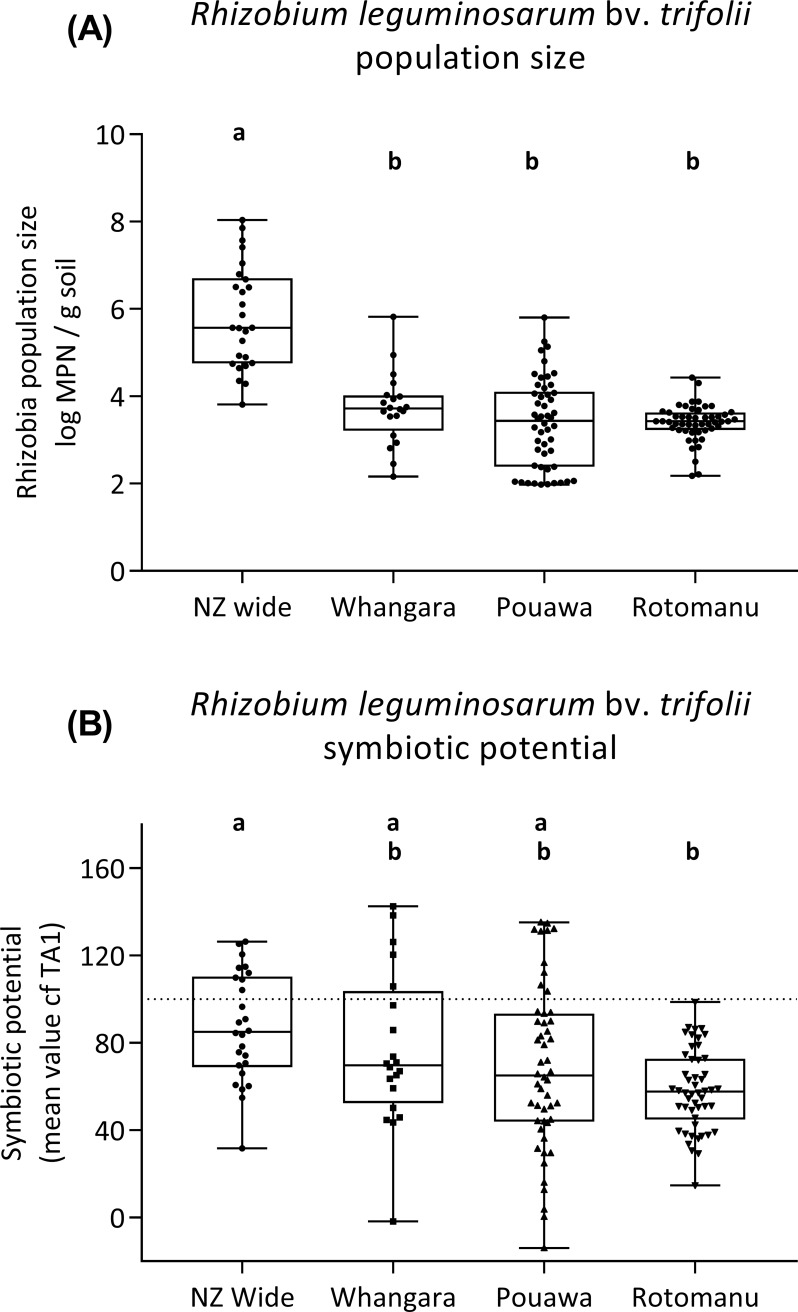
(A) *Rhizobium leguminosarum* bv. *trifolii* population size and (B) nitrogen fixing effectiveness (symbiotic potential relative to commercial *Rlt* strain TA1) in soils across different spatial scales. Boxes show median values and extend to the 25th to 75th percentiles. For 2B, the dashed line at 100% symbiotic potential = white clover growth when inoculated with the commercial rhizobia inoculant strain TA1. Treatments sharing the same lettering (*a* or *b*) have similar treatment means (Bonferroni comparison of means; α = 0.05).

The size of *Rlt* populations varied significantly among the sampling sites (*P*<0.001); the data are presented in Fig 2A and given in the supplementary file (S5 Table). For the survey of farms from across New Zealand (n = 26), the mean population size was significantly greater than for any of the other sample sets (*P*<0.05; Fig 2A), with an average of 5.89 (log_10_) *Rlt* per g of soil (= 777,585 *Rlt* g^-1^ soil). The mean values for *Rlt* populations collected at farm or field spatial scales did not significantly vary (*P*>0.05; Fig 2A).

**Fig 3:**
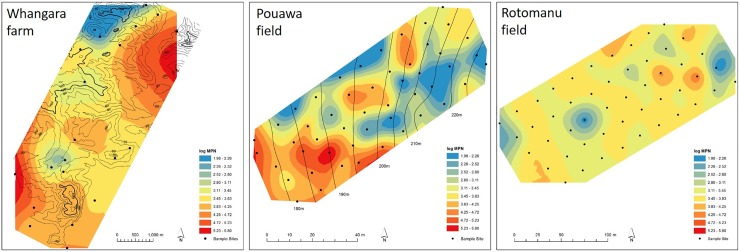
Spatial variation in *Rhizobium leguminosarum* bv. *trifolii* population size, (most probable number) based on nodule formation on *Trifolium repens* cv. Tribute. Black dots represent sampling spots based on GPS-marked points. Contour lines for Whangara and Pouawa are given; the field at Rotomanu had flat topography. MPN variation between samples was assessed using the RBK method in ARC-GIS.

Similar levels of variation in *Rlt* populations size were found for populations from across New Zealand, at Whangara, and within the field at Pouawa (*P*>0.05). However, the variation in MPN determined in the 50 samples from the field at Rotomanu was lower than that measured for any of the other sample sets (*P*<0.05). The level of variation in *Rlt* populations across these spatial scales (Whangara, Pouawa, and Rotomanu) are show in Fig 3.

### Effectiveness of *R*. *leguminosarum* bv. *trifolii* populations in pasture soils

All SP data for the sample sites are given in the supplementary file (S5 Table). The average SP of the *Rlt* populations is presented in Fig 2B. For each of the four sample sets, the average SP for was lower than that of the commercially available *Rlt* strain, TA1. However, there were many rhizobia populations collected from across New Zealand, at Whangara, or within the field at Pouawa, where SP’s ranged from 130 to 143%. In some cases, populations at Pouawa and Whangara had negative symbiotic potentials (-14% and -2%, respectively).

**Fig 4:**
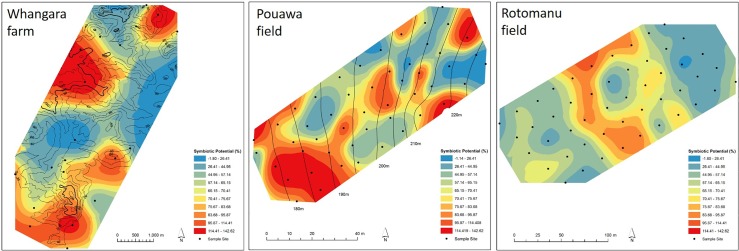
Spatial variation in *Rhizobium leguminosarum* bv. *trifolii* symbiotic potential (SP); i.e. efficacy at increasing the growth of *Trifolium repens* cv. Tribute. Assay conditions for clover growth had minimal nitrogen content, such that plant growth was directly related to N-fixation occurring through symbiosis. SP is calculated relative to growth of *T*. *repens* c.v Tribute inoculated with the current commercial *R*. *leguminosarum* bv. *trifolii* strain used in New Zealand, TA1 (= 100%). Black dots represent sampling spots based on GPS-marked points. Contour lines for Whangara and Pouawa are given; the field at Rotomanu had flat topography. Variation in SP between samples was assessed using the RBK method in ARC-GIS.

The large variation in the effectiveness of the soil rhizobia populations was notable. The SP of populations at Whangara, for example, ranged from 2% to 143%. Furthermore, variation in SP within the single hill country field, Pouawa, was greater than that in samples collected across New Zealand (*P* = 0.037). At Rotomanu, variation in SP within the field was lower than that measured for any of the other sample sets (*P*<0.05). These variations in SP, at different spatial scales, are evident in Fig 4.

### Relationship between size and SP of *R*. *leguminosarum* bv. *trifolii* populations

**Fig 5:**
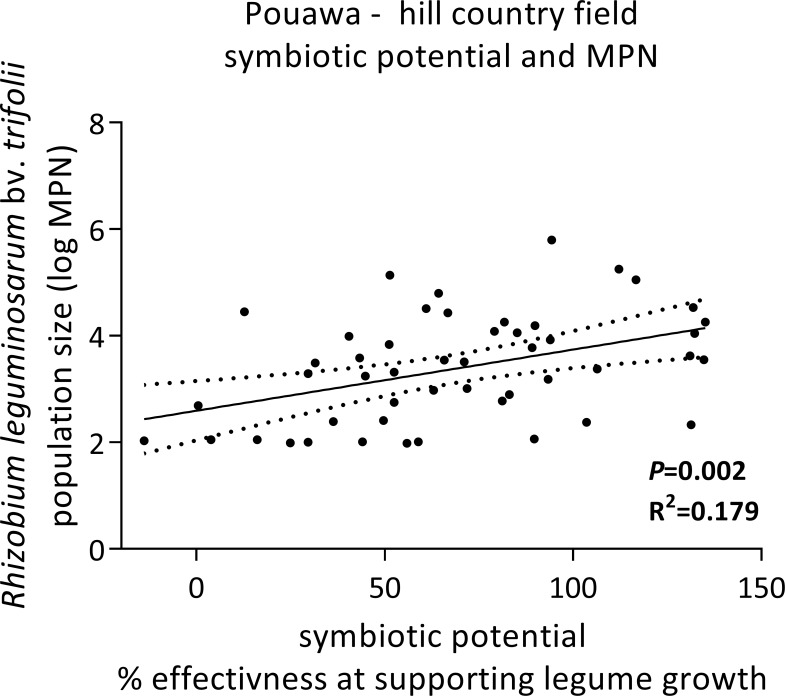
Relationship between the *R*. *leguminosarum bv*. *trifolii* population size and symbiotic potential of the legume-rhizobia symbiosis in 50 samples of soils collected from within a single field at Pouawa (hill country sheep and beef farm). Solid line is the linear regression model fit; dashed lines are the 95% confidence intervals.

No relationships were evident between the numbers and effectiveness of *Rlt* in the collection of soils from across New Zealand (R^2^ = 0.08; *P* = 0.145), at the Whangara farm (R^2^ = 0.004; *P* = 0.780), or the field at Rotomanu (R^2^ = 0.015; *P* = 0.390). At Pouawa, MPN and SP were linearly related (*P* = 0.002), though the association between the two variables was weak (Fig 5; R^2^ = 0.179).

### Correlations between size of *R*. *leguminosarum* bv. *trifolii* populations and soil properties

**Fig 6:**
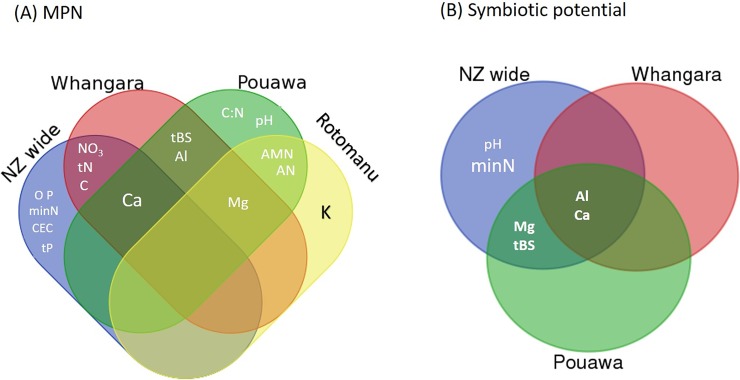
Venn diagrams showing similarity in properties of soils, at different spatial sampling scales, that have significant correlations with *Rhizobium leguminosarum* bv. *trifolii* population size (MPN’s; 6A), and symbiotic potential (6B).

Results of Pearson’s correlations between soil physicochemical properties and *Rlt* MPN values are given in Table 1. As the geographic scale of sampling changed, soil properties correlated with MPN changed. For most sites, only 2–3 soil properties correlated with MPN were shared (Fig 6A).

**Table 1:** Summary of Pearson’s correlations between *Rhizobium leguminosarum* bv. *trifolii* populations size (MPN values) and symbiotic potential with soil physicochemical properties in samples with different geographic ranges.

Most probable number								
**Variable**	**New Zealand**		**Whangara farm**		**Pouawa field**		**Rotomanu field**	
	**r**	***P***	**r**	***P***	**r**	***P***	**r**	***P***
NO_3_-N	0.7489	<0.0001	-0.605	0.0047				
Total P	0.6648	0.0002						
Mineral N	0.6251	0.0006						
CEC	0.5479	0.0038						
Total N	0.4663	0.0163	-0.5681	0.009				
Total C	0.4572	0.0189	-0.5328	0.0156				
Ca	0.3956	0.0454	0.5861	0.0066	0.573	<0.0001		
Olsen P	0.3895	0.0492						
TBS			0.6964	0.0006	0.6057	<0.0001		
Mg			0.508	0.0222	0.6117	<0.0001	-0.4473	0.0011
Al mg/kg			-0.4635	0.0396	-0.5333	<0.0001		
pH					0.4153	0.0027		
AN					0.3571	0.0109	-0.2824	0.0469
C:N					-0.3236	0.0219		
AMN					0.3067	0.0303	-0.335	0.0174
K							-0.3022	0.0329
Symbiotic Potential								
**Variable**	**New Zealand**^**1**^		**Whangara farm**		**Pouawa field**		**Rotomanu field**	
	**r**	***P***	**r**	***P***	**r**	***P***	**r**	***P***
pH	0.6744	0.0002						
TBS	0.6284	0.0006			0.4196	0.0024		
Al mg/kg	-0.4375	0.0325	-0.6177	0.0037	-0.2984	0.0373		
Mineral N	-0.4195	0.0329						
Ca	0.4187	0.0332	0.6026	0.0049	0.3152	0.0258		
Mg	0.4042	0.0406			0.4087	0.0032		
Rainfall	-0.661	0.0002						
Temp (min)	-0.394	0.046						

TBS = total base saturation, CEC = cation exchange capacity, AN = available N, AMN = anaerobically mineralisable N. All cations as me/100g.

^1^meteorological data (rainfall and temperature) collected for NZ wide sampling only.

Across the NZ wide and Whangara farm sample sets, strong and significant correlations between MPN and soil NO_3_-N content were determined. While this was negative for Whangara (r = -0.605), i.e. MPNs increased as NO_3_-N declined, the reverse was true for the NZ wide sampling (r = 0.789; Table 1). Across NZ, soil P fertility (total and Olsen extractable P) was positively correlated to MPN values, but P was not linked with *Rlt* population sizes at any of the other sampling scales (*P*>0.05). Meteorological properties (temperature, rainfall, solar radiation etc.) had no correlation with MPN at the NZ sampling scale.

### Correlations between *R*. *leguminosarum* bv. *trifolii* symbiotic potential and soil properties

The summary correlation (Pearson’s r) and P values are given in Table 1. Overall, far fewer correlations between soil physicochemical properties and *Rlt* symbiotic potential were found compared with the MPN data (Table 1; Fig 6). At the New Zealand scale, strongest correlations were with pH, rainfall, and TBS, none of which were correlated with MPN (Table 1). However, at Whangara, strong positive correlations with Ca and both symbiotic potential (R^2^ = 0.60) and MPN (R^2^ = 0.59) were present. With the exception of Rotomanu, soil Ca and Al contents were both positively correlated to symbiotic potential (*P*<0.05; Table 1). Soil properties associated with symbiotic potential that are shared or unique among sites are given in Fig 6B.

## Discussion

### Rhizobia population size

Across New Zealand the average size of the *Rlt* population was 6.6 x 10^5^, however this ranged from a maximum of 1.1 x 10^8^ to a minimum population size of 6.5 x 10^3^ rhizobia per gram of soil. These values fall within ranges reported in previous investigations [11–14,27]. A goal of this work was to identify the extent to which these values vary, and particularly if soil properties can explain a portion of the variation. Given the soils were all collected from under long term pasture, and contained legumes as a component of the pasture sward, we could remove influence of previous land use from the analysis (e.g. legacy effect of previous crop or landuse). By assessing MPNs across a range of sites (26 in this case), we aimed to be able to cover a range of potential variables in soil physicochemical properties that could be associatively linked with MPN [25].

The closest correlation among soil properties to MPN was with NO_3_-N; this relationship was positive and strong (r = 0.749; *P*<0.0001). However, given the outcome of effective rhizobia-legume symbiosis is fixation of atmospheric nitrogen, it is likely this correlation indicates that increased soil N-fertility is an emergent outcome of greater rhizobia populations, not a driving factor controlling population size in this case. The exception would be at high soil NO_3_-N concentrations, where nodule formation by the legume is suppressed [28] and potentially reducing soil rhizobia populations over time.

The other significant correlation was *Rlt* population size with soil P content. White clover has a high requirement for P [29], and insufficient soil P may reduce clover growth and result in lowered host-capacity (root biomass) to support *Rlt* populations in the field. At farm scale, or within field, this relationship would be unlikely to manifest as variation in phosphate as, at these scales, variation in soil P would be small. Studies conducted at field-level, for example, are more likely to be constrained in the range of physicochemical properties due to soil type. Indeed, soil properties associated with ‘field level’ variation in MPN values had virtually no overlap with those occurring across New Zealand. These results show the importance of appropriate interpretation of rhizobia-associated data collected at different spatial scales.

### Symbiotic potential

The presence of large naturalised rhizobial populations in New Zealand’s pastoral soils has led to the suggestion that inoculation of seed is not necessary in the majority of situations where white clover is grown [11]. As such, a key result of this study was fining no evidence for a relationship between *Rlt* population size and symbiotic potential at all sites except Pouawa. As such, use of of MPN from pasture soils as indictors of nitrogen-fixation performance should be reconsidered.

At the NZ scale, symbiotic potential was most closely linked to soil pH, TBS, and rainfall (Table 1). With the exception of TBS at Pouawa, these factors were not correlated with symbiotic potential for the other sample collections. This outcome was likely underpinned by the spatial scale at which these variables effectively vary. Surprisingly similar links were found in a study of *R leguminosarum* bv. *viciae* on field pea. Across 114 soils, pH and average rainfall accounted for 17% in the variation of symbiotic potential [25].

Across New Zealand, the average symbiotic potential was 87.3%, and the standard deviation 24.8%. The clover-rhizobia symbiosis formed with less effective naturalised strains will result in less than optimal N fixation rates. However, in most situations, poor N_2_-fixation is not recognised because the effects on plant growth are ‘sub-clinical’ on all but the most deficient soils [30].

The populations of rhizobia in the soil sampled most likely originated from commercial, and therefore presumably highly effective, inoculant strains applied over many decades [11]. As such, the results here indicate that there has been an erosion of the rhizobia genetic resources over time [25]. This may have occurred through a number of mechanisms, including exchange of genetic material with the microbial community, colonisation of the sites with ineffective strains, etc. However, the associations between environmental stress (pH and moisture) and rhizobia fitness across studies [16,25], indicate both edaphic and environmental factors may be driving genetic instability in rhizobia populations [31]. This could be tested by comparing variation in the genetic structure of the rhizobia communities across these sites, with the hypothesis that soils with greater environmental stress (pH, rainfall) have greater variation in rhizobia genetics at the population level.

Negative symbiotic potentials were found in a few samples. In these instances, the net effect of deleterious microflora outweigh the importance of beneficial symbionts in these soils. Recent surveys of pathogenic microorganisms and nematodes in New Zealand pastures have revealed surprisingly large impacts of disease on white clover growth [32,33]. The inoculation of soils harbouring these pathogen-enriched communities into clover growth assays can result in poor outcomes for clover seedlings.

### Spatial variation in symbiotic potential is high

Variation in symbiotic potential of *Rlt* populations was high even at small spatial scales. Indeed, the 0.8 ha field at Pouawa had greater variability than the 26 sites spanning ca. 1000 km in distance across New Zealand (*P* = 0.037). Thus, when collecting information for the purposes of understanding rhizobia ecology or predicting rhizobia requirements for legumes (e.g. inoculation of seed), the consideration of extent of spatial variably is vital. Standard field sampling, such as that carried out for analysis of soil fertility, typically involves collection of numerous subsamples that are mixed into a single composite sample. This ‘field average’ effectively masks underlying spatial variation within the field; areas with low or ineffective rhizobia populations will not be identified. If these areas are not supplied with sufficient rhizobia, variable or poor establishment and performance of legumes may result. Given the fundamental importance of legume and BNF in pastoral farming systems [4], and difficulties experienced in legume establishment in hill country soils [5], ensuring legumes are matched with an effective nodule-forming and nitrogen fixing (nod^+/^nif^+^) strain is important. As the cost of accurately mapping populations in soils is high, and given that high variability in effective populations is expressed at field level, a precautionary approach is to supply rhizobia where clover is being sown.

Notably, the field site at Rotomanu had the lowest variability in MPN and symbiotic potentials. This may be due to the uniformity of the soil type, the flat topography of the field, or the uniformity in underlying soil properties.

### Improving soil rhizobia resources

If improving the rhizobia present in New Zealand pastoral soils is necessary to lift the potential productive capacity of these agroecosystems, a key step is identifying opportunities to build genetic gain in rhizobia populations. Historically, this has been achieved through delivery of highly effective strains of rhizobia into soils, usually on the surface of clover seed at the time of sowing [11]. However the efficacy of this method is limited by a number of factors including poor rate of survival of rhizobia on some commercially-treated legume seed [34,35].

An alternative method to establish rhizobia into pastoral soils is through direct soil inoculation. This may coincide with delivery of lime, another fertiliser, or during sowing other seed such as ryegrass. Such methods may overcome many of the issues associated with use of clover seed as the primary carrier for rhizobia (see above), and provide a more uniform and potentially higher population levels of rhizobia. However, competition with existing field populations may impact on the success of this approach, or others, for establishing microorganisms into soils. As such, knowledge on the ecology of the ecosystem, with a view towards identifying best opportunities for establishment of new rhizobia is critical (invasion ecology). Consideration should also be given to the potential for the symbiotic potential at sites to decline over time. As discussed previously, shifts in the population genetics of rhizobia may be driven under conditions of edaphic or environmental stress. The targeted use of rhizobia adapted to specific soil stress, e.g. desiccation [16], may not only result in better strain survival in the field, but may provide a more genetically stable population over time.

## Conclusions

Appropriate consideration of spatial influences must be incorporated into field based rhizobia studies and analysis of such results. In particular, underlying edaphic and environmental variables associatively linked with variation in rhizobia population size or efficacy differ depending on the spatial scale of sampling. If these factors are not controlled or considered during interpretation of the findings, then recommendations extending from these studies maybe misinformative. Understanding of spatial variability in rhizobia populations is essential to providing accurate advice regarding needs to add new rhizobia to farming systems such as via seed inoculation. Field-average data obscure local spatial variation, masking local areas harbouring either effective or ineffective populations. The loss in symbiotic performance of field populations could be linked with accelerated genetic change caused by environmental stress. This requires formal testing using a population genetics approach. However, it may offer an opportunity to target specific stress-adapted rhizobia to farming systems, thereby building genetic gain within existing rhizobia populations.

## Supporting information

S1 Table Soil locations, type, physicochemical properties, for the 26 New Zealand-wide samples. (XLSX)

S2 Table Sample locations and soil physicochemical properties for the 20 soils collected from the hill country sheep and beef farm, Whangara. (XLSX)

S3 Table Soil physicochemical properties for the 50 soils collected from within a single field on a sheep and beef farm, Pouawa. (XLSX)

S4 Table Soil physicochemical properties for the 50 soils collected from within a single field on a dairy farm, Rotomanu. (XLSX)

S5 Table *Rhizobium leguminosarum* bv. *trifolii* population size (log MPN) and symbiotic potential values for the four datasets. (XLSX)
